# Hydrate of Chloral as an Anæsthetic

**Published:** 1870-01

**Authors:** 


### EDITORIAL DEPARTMENT.

*Hydrate of Chloral as an Anœsthetic.*—A great deal of interest is manifested in this anæsthetic compound, and there is scarcely an exchange received which does not contain some notice of its nature and properties.

Chloral, from which the hydrate is obtained, is formed by passing chlorine gas through anhydrous alcohol, with subsequent distillation. The hydrate takes the form of white crystals, which are very soluble and readily absorbed when they are administered by the mouth or by subcutaneous injection. The manufacture of chloral is attended with difficulty and expense. Well dried chlorine gas is passed through pure anhydrous alcohol as long as it is absorbed, which process consumes many hours, and the vessel containing it must be kept cool in the first stages of the operation, but in the following stages the temperature must be gradually raised until the contents boils. Absolute alcohol must be employed in the preparation of chloral, for if it is diluted, instead of chloral being formed we have aldehyd, acetic acid, and hydrochloric acid. Care is also necessary to prevent the generation of such compounds as chloride of carbon and chlorous acetylene, which are dangerous. After the passing of the chlorine gas through anhydrous alcohol, the crude product is mixed with three times its bulk of oil of vitriol, and distilled by means of a gentle heat; which operation is often repeated several times and the product distilled over quick-lime.

This is by no means a new anæsthetic, as it has been known in chemistry about thirty-eight years. Liebig having discovered chloral (from which the hydrate is made by the simple addition of water,) in 1830, but, as Dr. B. W. Richardson states, the introduction of it into medicine is a fact of the present year, its introducer being Liebreich of Berlin.

Hydrate of chloral then is a chemical combination of water and chloral, the process for the generation of this latter having been described above. It is expensive, as was before remarked, because one pound of alcohol yields less than an ounce of chloral. When the chloral is generated it has the appearance of a colorless, oily liquid, with a pungent odor, and is an irritant to the skin. The chloral is now mixed with a small quantity of water, and on applying heat it solidifies, the product being a white crystalline substance known as the hydrate of chloral—the an-

æsthetic in question. The crystals are rhombic, contain 90 parts of chloral, as in their formation eight parts of chloral combine with one part of water, forming nine parts of hydrate of chloral. It volatizes readily without decomposition although in a solid form, resembling in this respect camphor. Water, alcohol and ether dissolve it. The manner in which hydrate of chloral is administered, is in solution with water, either by subcutaneous injection or by the mouth directly into the stomach. Dr. Richardson tells us that the best solution is made by mixing one grain of the hydrate with two of water. and he also affirms that it can be administered, to some extent, by inhalation. Dissolved in an excess of water the taste is agreeable, with the odor of a ripe melon.

Administered to human subjects, doses varying from thirty to sixty grains caused unconsciousnes to pain and a profound sleep lasting over several hours The sleep is quiet and gentle, induced without distress, and leaving no other symptom behind, except nausea which is occasionally experienced after recovery. In administering this agent to the human subject, Dr. Richardson, states that allowance will have to be made not only in relation to size and weight, but to obesity or leanness, to natural habit and actual state of body in respect to sensibility. In administering it to lower animals, Dr. R. also says, there is a common rule in respect to weight of animal and dose of hydrate; thus an animal weighing, say three ounces, requires one grain of the narcotic to be brought fairly and fully under its influence. If the proportion of dose be beneath this, the symptoms will be imperfectly developed; if it be much exceeded the symptoms will end in fatal sleep. Liebreich claims to have produced sleep which lasted from five to fifteen hours, with from 25 to 30 grains of the hydrate, but experiments made with an American preparation by Dr. Hasket Derby were not so satisfactory, more of the agent being required and a longer time to induce sleep, as well as the sleep continuing for a much less time and in none of the cases being of a longer duration than four hours, although the quantity administered was as much as 75 grains.

This anæsthetic agent appears to act more promptly when subcutaneously injected than when administered directly by the mouth; and as chloral dissolved in water is slightly caustic, it cannot be administered by the mouth when there are lesions of mucous membrane, or ulcerated tracts of intestinal canal.

While Liebreich considers chloral an anæsthetic of some power, though inferior to chloroform and ether, Dumarquay, a French writer, stated to the Academy of Sciences, that as a result of experiments on animals and men, he considers it no anæsthetic, as the skin is sensitive no matter how great the intensity of the sleep. He finds it, however, the most rapid of hypnotics, and draws the following deductions: "1. Chloral has a marked hypnotic action, especially on feeble and debilitated individuals. 2. The duration is in direct agreement with this feebleness. 3. The sleep provoked is generally calm, and is exerted only in those patients who are a prey to lively pain, This leads one to counsel its use in those diseases where it is desired to bring on sleep and muscular relaxation. 4. Finally, this agent can be given in doses of 1 to 5 grammes without determining any accident."

On the other hand M. Bouchut, before the same Academy, agrees with the views of Liebreich, and asserts that "chloral exerts powerful sedation upon both motor and sensitive nerves, when employed in the crystallized form and free from impurities; that the dose should not exceed five grammes (77.16 grs.) for adults, or one or two grammes for children. He condemns as dangerous its use by hypodermic injection, and states that it is more rapidly absorbed by the rectum than by the stomach. He agrees with Leibreich in attributing its action to its conversion into chloroform. The sleep induced by it is sometimes attended by an agreeable intoxication. Hyperæsthesia is rare under its influence, the usual result being anæsthesia, varying in degree according to the strength of the dose. In its therapeutic relations, M. Bouchut pronounces chloral to be the most valuable sedative known in the violent pain of gout, of nephritic colic, or of dental caries—the best of anæsthetics to be given by the stomach; and the quickest and surest remedy in aggravated chorea, to quiet the restlessness which is the most serious symptom of that disease."

M. Leon Labbe states as the result of his experiments:

"1. Chloral, when introduced in sufficent quantity into the blood of an animal, produces anæsthesia, but without that preliminary condition of excitement which obtains with chloroform.

2. When introduced into the alimentary canal, or under the skin, it produces first sleep, and then anæsthesia, but in a lesser degree than when directly introduced into the blood.

3. In these cases there is some slight irritation, but nothing to be compared with hyperæsthesia."

